# Back to live: Returning to in-person engagement with arts and culture in the Liverpool City Region

**DOI:** 10.3389/fpsyg.2022.1011766

**Published:** 2022-10-26

**Authors:** Antonina Anisimovich, Melissa Chapple, Joanne Worsley, Megan Watkins, Josie Billington, Ekaterina Balabanova

**Affiliations:** ^1^Department of English, University of Liverpool, Liverpool, United Kingdom; ^2^Department of Primary Care and Mental Health, University of Liverpool, Liverpool, United Kingdom; ^3^Institute of Health and Social Care, London South Bank University, London, United Kingdom; ^4^Department of Communication and Media, University of Liverpool, Liverpool, United Kingdom

**Keywords:** arts engagement, COVID-19, cultural industry, mental health, wellbeing, digital provision

## Abstract

On July 19th 2021, the UK government lifted the COVID-19 restrictions that had been in place since March 2020, including wearing masks, social distancing, and all other legal requirements. The return to in-person events has been slow and gradual, showing that audiences are still cautious when (and if) they resume engaging in arts and culture. Patterns of audience behavior have also changed, shifting toward local attendance, greater digital and hybrid engagement, and openness to event format changes. As the arts and cultural industry recovers from the pandemic, it is important to adopt an audience-oriented approach and look at the changing patterns of engaging in arts and culture. This study aims to better understand the impact of the pandemic on the patterns of cultural and arts engagement. Eight qualitative interviews were conducted to explore the changes in arts and cultural engagement since the restrictions were lifted, focusing particularly on the audience’s experiences of returning to in-person arts and cultural events in the Liverpool City Region (LCR). Using framework analysis, three themes were identified from the data: *The new normal: reframing pre-pandemic and pandemic experiences of arts and culture*, *Re-adjusting to in-person provision*, and *Moving forward: online and blended provision*. The findings show that the pandemic altered the ways that people engage in arts and culture. The “new normal,” a blend of pandemic and pre-pandemic experiences, illustrates how the pandemic has highlighted and reconfigured the importance of arts and culture, in terms of personal and cultural identity. Resuming in-person engagement after a long break, participants noted that they were able to feel more like themselves again. Arts and culture were perceived to be beneficial in rebuilding personal resilience and confidence. Engaging in arts and culture, following the isolating experience of the pandemic, has also helped participants feel reconnected to others through their shared experiences. Finally, the findings suggest that online provision remains vital for many, ensuring wider inclusivity, particularly for vulnerable audiences. At the same time, it is important to acknowledge the barriers to online inclusion and the possibility of this resulting in a growing digital divide.

## Introduction

As documented in numerous studies across the globe, the COVID-19 pandemic had a significant impact on mental health and wellbeing (Daly et al., 2020; Yamamoto et al., 2020; Niedzwiedz et al., 2021). There is some evidence to suggest that the pandemic has made pre-existing inequalities even more prominent. Some of the demographic groups whose mental health was disproportionally affected by the pandemic include young people (Singh et al., 2020; Yamamoto et al., 2020; Kwong et al., 2021), women (Daly et al., 2020; Niedzwiedz et al., 2021), black and ethnic minorities (Proto and Quintana-Domeque, 2021), as well as people from more socially disadvantaged backgrounds and those with pre-existing mental health conditions (Kwong et al., 2021; O’Connor et al., 2021). Both during and immediately after the lockdowns, people reported feelings of isolation, anxiety, COVID-19-related sleeplessness, as well as work and study difficulties as factors that had the most adverse effect on their mental health and wellbeing (Yamamoto et al., 2020).

Numerous benefits to engaging in arts and culture have been widely reported, including personal development, social cohesion, community empowerment, improved local image and identity, and promotion of imagination and vision (Matarasso, 1997). There is an established and well-researched link between regular engagement in arts and culture and mental health and wellbeing (Matarasso, 1997; Daykin et al., 2018; Fancourt and Finn, 2019) and it is that aspect of the value of arts and culture in people’s lives with which this paper is principally concerned. There is also a growing body of evidence on the mental health and wellbeing benefits of engaging in arts during the pandemic. For example, engaging in hobbies such as reading and music has been associated with decreased depression and anxiety levels and increased life satisfaction (Bu et al., 2021; Cabedo-Mas et al., 2021). Higher levels of engagement in arts and culture also had a positive impact on people’s resilience and ability to cope with adverse situations (Keisari et al., 2021).

During the COVID-19 pandemic, in-person engagement was affected by the national lockdowns that introduced a number of restrictions. The pandemic had a serious impact on the arts and culture industry since many organizations had to suspend their in-person provision and move their activities online. On the 19th of July 2021, the UK government lifted the COVID-19 restrictions that had been in place in some form since March 2020, including wearing masks, social distancing, and all other legal requirements. As the arts and culture industry recovers from the effects of the pandemic, it is important to explore the changing patterns of engaging in arts and culture and adopt an audience-oriented approach moving forward (Radermecker, 2021).

As Sedgman (2016) notes in her text “Locating the Audience,” “audiences” are never a simple monolith group. They can no longer be perceived as a homogenous group of passive receivers of arts and culture (Bishop, 2012) but instead are to be viewed as a dynamic group able to actively “engage, to attend, to co-create, to participate”. The complexity and variety of audiences’ experiences also makes them a somewhat difficult group to study. However, despite these challenges linked to audience research, it remains crucial to “hear audiences out” (Sedgman, 2016) and give them voice. As Kershaw (2001) suggests, engaging in arts and culture always holds a democratic potential, which is why the audiences’ experiences should never be reduced to a passive consumption of arts.

Inevitably, the COVID-19 pandemic and the subsequent closures of arts and culture venues made the discussion of audience participation topical again. As lockdowns and the digitization of arts provision have transformed the interaction between arts and culture providers and their beneficiaries, it has become even more important to understand audiences and how they engage in arts and culture particularly during such turbulent times as a global pandemic. Focusing on the variety and complexity of the lived experiences of arts and culture engagement and the benefits derived from it during the pandemic, this paper uses the concept of audiences and beneficiaries interchangeably.

As recent studies demonstrate, the return to in-person events has been slow and gradual, showing that audiences are still cautious when they resume engaging in arts and culture (The Audience Agency, 2021). Moreover, the patterns of audience behavior have also changed, shifting toward more local attendance, greater digital and hybrid engagement, and openness to event format changes (The Audience Agency, 2021). Remaining domestic COVID-19 regulations came to an end in February 2022 but the changed patterns of engagement could inform new ways of moving forward and help evaluate the impact of the pandemic on the arts and culture sector.

This paper undertakes the crucial task, therefore, of examining this transitional period in order to understand audience concerns and expectations moving forward. It looks at the specific changes in arts and cultural engagement occurring after the lifting of restrictions in the UK, focusing on the audiences’ experiences of returning to in-person events, as well as their engagement with the continued hybrid and online arts and cultural provision in a specific geographical location–the Liverpool City Region (LCR).

Liverpool City Region has one of the highest concentrations of culture in the UK and the largest clustering of museums and galleries outside London. Culture, arts, and creativity are central to the city’s identity (Belchem, 2006), and cultural capital is vital for the city region’s economy. At the same time, the region had some of the poorest mental health outcomes in the UK prior to the pandemic and the pre-existing regional inequalities between the North and the rest of the country were exacerbated by the pandemic, resulting in a significant decrease in mental and financial wellbeing in the North West (NHSA, 2021). In LCR almost one in five (19.4%) of the population aged 16+ has a common mental disorder, compared to the national average of 16.9% (Liverpool John Moores University, Public Health Institute, 2021).

The LCR is home to several successful programs harnessing arts for mental health care through partnerships between culture and health providers (Billington et al., 2013; Burns, 2017). During the pandemic, the arts and cultural organizations in the region proved to be a “lifeline” for many people, offering a much-needed way to combat isolation and stress (Worsley et al., 2022). This combination of factors highlights the LCR as a distinctive case study for identifying how arts and culture supported mental health and wellbeing following the conclusion of lockdown.

## Materials and methods

Qualitative semi-structured phone and online video call interviews were conducted in July and August 2021 following the full easing of COVID-19 restrictions in England. Interviews reflect on the experiences of in-person events, when there were no legal requirements to maintain any levels of restrictions. However, despite the lifting of national restrictions, during this time all venues had different safety measures in place (for example, some civic venues had restrictions in place until September 2021). Moreover, the criteria for arts and cultural engagement were wide and included a variety of experiences, ranging from live music performances to arts exhibitions and participation in a choir. This has inevitably translated into a variety of participant experiences; the current study captured a wide range of engagement with arts and culture in the LCR.

### Ethical approval

Ethical approval was received from Liverpool University Research Ethics Committee (reference number 7994).

### Participants

Eight qualitative interviews (seven female and one male participant) were conducted. Participants were recruited *via* an advert disseminated through social media (project Twitter account) and published on the University of Liverpool website. The advert was also circulated by arts and cultural organizations in the LCR. Participants were interviewed *via* Zoom or by phone. All contacted participants agreed to take part in the study and provided informed written consent. For all eight participants, this was the third and final interview of the study. The current study builds upon findings of wave one and two data (Chapple et al., under review) that focus on the experiences of lockdown and during the initial restrictions easing. The longitudinal aspect of the wider study ensured a wide variety of data and explains why no further recruitment was needed for wave three.

### Data collection

This study was conducted throughout July and August 2021, after the lifting of restrictions in England on 19th July 2021. The interviews were conducted using a prepared topic guide that included questions about recent engagement in arts and culture in the LCR, particularly the return to in-person events, as well as online and hybrid engagement. Interviews duration ranged from approximately 20 to 45 min. The interviewer produced field notes during the interviews. The interviews were audio recorded and then transcribed verbatim using Otter.ai software. All interviews were conducted by the first author, a female postdoctoral researcher with prior training in qualitative and quantitative research methods.

### Analysis

Interview data were analyzed in NVivo version 12 using framework analysis (Ritchie and Spencer, 1994). Framework analysis was chosen because it allows for a combination of deductive and inductive data analysis particularly helpful for analyzing and comparing complex data sets. The procedure outlined by Gale et al. (2013) was followed. Familiarization was reached *via* transcription, re-reading of the transcripts, and creating a memo file summarizing the key points of each transcript. Memo notes were shared with the wider team. Codes and categories from the previous waves of the study were used as an initial organizational framework to sort the data and identify any changes from previous data.

Three transcripts were read, coded independently, and discussed by two researchers to ensure inter-coder reliability. After agreement on codes was reached, all transcripts were coded line-by-line within NVivo. All codes were then grouped into categories, focusing on the changes observed in this dataset compared to previous waves (Chapple et al., under review), producing a working analytical framework. This framework was then applied to all transcripts through indexing. Following a discussion with the wider team, the themes and subthemes were edited and refined, resulting in a finalized analytical framework.

## Results

Three themes were identified from the interview data: *The new normal: reframing pre-pandemic and pandemic experiences of arts and culture*, *Re-adjusting to in-person provision*, and *Moving forward: online and blended provision.* Each theme comprised a number of subthemes (see Table 1).

**TABLE 1:** Overarching themes and subthemes.

Themes	Subthemes
The new normal: reframing pre-pandemic and pandemic experiences of arts and culture	● *Reconnecting to the sense of self* ● *Rebuilding confidence and resilience* ● *Reconnecting with others through arts and culture* ● *Reframing cultural identity (local and global blurred)*
Re-adjusting to in-person provision	● *Varied experiences of in-person events after restrictions lifted* ● *Taking risks: varying levels of safety* ● *Managing change during the transitional period*
Moving forward: online and blended provision	● *Navigating online content* ● *Considerations around online inclusion*

### The new normal: Reframing pre-pandemic and pandemic experiences of arts and culture

#### Reconnecting to the sense of self

This subtheme reflects on arts as a way of reconnecting with one’s identity, where the latter has been shaped by lifelong engagement in arts and culture. As previous research shows, arts and culture are ingrained in personal narratives for many participants, making the limited access to arts and culture during lockdown particularly challenging and leading to a certain sense of loss of self-identity (Chapple et al., under review). During the transitional period of returning to live events, many participants have not yet been able to return to their pre-pandemic levels of engagement even as the restrictions were lifted. The sense of loss of arts and culture as a vital part of their identity remains a challenge:

*I still love looking at stuff and planning stuff. I haven’t done that. Well, I stopped doing that completely, which is*… *A little part of my life has changed. Hopefully, it will come back? Definitely* (P5).^1^

Furthermore, participants also noted the long-term detrimental effect of the pandemic on their motivation, particularly when planning an event out, even though this used to be usual for them pre-pandemic:

*So those are the things I’ve instigated, but everything else has been somebody else and that’s not usual for me. Normally, I love looking at what’s on and then planning and going, I just haven’t done it. I mean, I’m hoping to come back* (P5).

Despite the challenges mentioned above, as COVID-19 restrictions were lifted, some participants were able to return to in-person provision and thus, reclaim a part of their identity through arts and culture:

*Those cultural opportunities, they were the things that were motivating me to be able to be more myself* (P6).

#### Rebuilding confidence and resilience

As previous data show, engaging in arts and culture during the pandemic helped participants cope with isolation and stress (Chapple et al., under review). Data collected after the restrictions were lifted highlight the crucial role of arts and culture in instilling confidence and rebuilding resilience. As participants were able to engage in arts and culture in-person again, they reported that this experience, even if stressful at first, allowed them to gradually regain confidence in relation to attending other in-person events:

*Just doing it once increased my confidence. [*…*] I just think it’s a question of just having a little bit of faith* (P5).

Furthermore, taking this kind of risk helped participants become more resilient, since their capacity to resume in-person arts and cultural engagement provided a positive example of how they were coping with the difficulties of the pandemic during this transitional time:

*I think it’s really been a good thing for me because it’s sort of got me into a better place, I think, to be able to handle what’s going to be a sort of unusual state of things to come, you know, and to sort of make that transition. I think it’s really helped actually* (P6).

This participant also provides an interesting perspective on risk-taking. During the pandemic, risk was reduced to issues of health and life, while previously it could also be perceived as something positive and humanizing:

*So in a way, some of these cultural things have actually helped me to be in a state that involves more risk in lots of different ways. It could just be that sense of an internal taking a risk. I don’t mean to your health, I mean, you know, that sort of uncertainty type thing. And they’re really important things to feel and to do. Because they’re the things that make you human* (P6).

#### Reconnecting with others through arts and culture

Participants’ excitement in relation to a shared experience was prominent. Many took this as an opportunity to reconnect with their personal networks. Here, a participant talks about going to a museum in Liverpool together with a lifelong friend that they had not seen for a long time:

*On Saturday, I’ve got a friend from Yorkshire. We used to go to school together. She lives in Yorkshire now. But she’s coming over on Saturday to come back to Liverpool for the day and we’ll go into the music thing because there’s an exhibition about Frankie Goes to Hollywood and they were our youth really* (P4).

Sometimes, participants gave precedence to socializing while engaging in arts and cultural activity, so that the experience of arts and culture itself was not the main priority:

*That one I didn’t enjoy, I didn’t necessarily enjoy the show. What I enjoyed was the experience of being out with friends again* (P8).

Participants highlighted the emotional aspect of being together again, as well as seeing other people enjoying arts and culture:

*It was really lovely to see people back on the streets, enjoying arts and culture again, that gave me a buzz*… *It just felt a bit of normal again, as well, seeing people kind of experiencing different things* (P7).

Some participants noted that socializing with people face-to-face has been a highlight of returning to in-person provision:

*I think being with people topped the lot* (P3).

As in the earlier interviews (Chapple et al., under review), participants affirmed that engaging in arts and culture during the pandemic helped them feel connected to others:

*We had a sort of joint Zoom of the choir with another choir. So that was really, that was a lot of fun. And it helped us I think, to feel connected, you know, because it was with other people doing singing, like we do. We were really pleased to be able to get in touch with them in that way* (P3).

Resuming in-person events was also an opportunity for some to meet new people and make new connections with others while enjoying arts and culture together in a shared space. This is an experience that many reported to be difficult to replicate online:

*Sometimes when you are just in a place and you start chatting to somebody about stuff, you know? [*…*] And it’s just nice to have a connection with somebody* (P4).

As the restrictions were lifted in July 2021, new opportunities emerged to resume in-person events without social distancing measures. This gave the participants an opportunity to be physically closer to other members of the audience. Some participants reported that this allowed them to feel more “normal,” closer to the usual pre-pandemic experience of a live event:

*And because they were in big tents and chairs were close together and stuff, that did feel like a proper night out, that did feel more of an event, of an atmosphere of everybody being in you know, sort of all there together. There was no distance in between the chairs or anything like that. It was literally you were all just sitting together. And that was really nice, and more what you would expect* (P4).

While some enjoyed the increased feeling of togetherness facilitated by being in a shared space together, many reported that social distancing measures and larger better-ventilated spaces were preferable because of safety concerns. While the participants missed mass events, not all of them would have felt comfortable at more crowded events at this time point. Among other concerns, this participant reported some feeling of awkwardness because of being in isolation for so long:

*That idea of like, you’ve gone so long without interaction* (P8).

#### Reframing cultural identity: Local and global identity blurred

As participants returned to in-person events, they continued to express strong appreciation for local culture, particularly for the cultural scene in the LCR:

*We are blessed, because we’ve got a pretty good cultural offer in the Liverpool City Region. I know it’s mainly based around the Liverpool city centre, but there is stuff in the Wirral, I live across the water. There’s so much on the doorstep, it is great. And it’s dead easy to access, and it’s really important* (P1).

As participants stated, the pandemic made them appreciate arts and culture even more, making them more aware of the opportunities that they used to take for granted:

*In the past, if they’ve not been local or they’ve not been in a place where I’ve been staying, I might have just let them pass me by unless I really desperately wanted to see them. Now I just think I’m lucky, I haven’t lost my income, you know I’m still working, I can afford it financially. [*…*] So this is my break now, this is the thing for me to do* (P4).

Some noted that the pandemic had made the vulnerable position of many arts and cultural institutions more visible, and participants were now more aware of the need to support local venues:

*I just think you’ve got to do things now. You know you don’t know how long things are going to be around and if you don’t support things, they might close down* (P4).

Along with supporting local institutions, some participants shared that they were happy to travel for arts and culture after the easing of restrictions as a way of catching up on the opportunities missed during the pandemic:

*When I went to Coventry, I saw three shows, because it was a city of culture this year, and I’ve got to go and support it. [*…*] Because of the pandemic, I sort of made a commitment to myself that you’ve got to you know, you’ve got to do things when you can and take advantage* (P4).

Many reported that the pandemic had in a way blurred the divide between the local and the global arts and cultural providers, offering new opportunities to create connections with likeminded people globally, as well as providing access to content worldwide:

*I’m sure there’ll be things that will happen through FaceTime and Zoom and whatever. They will continue because it gave access to people. I mean, gave you access to people from all over the world that you never would have had. So that’s brilliant*.

<…>

*I thought it was really lovely to be working collaboratively with another choir, even though there were just three of them. It was just fabulous to be connected with another group. Such a group as well. So we’re hoping that in future, because we all enjoyed it, we hope in the future, we might be able to collaborate more on future things* (P6).

### Re-adjusting to in-person provision

#### Varied experiences of in-person events after restrictions lifted

Due to many factors, the return to in-person events proved a challenging, even if exciting adjustment for the audience. For some, this experience highlighted how different life was during the pandemic, resulting in them almost “forgetting” how to engage in live events. Others felt so accustomed to online provision that it felt strange to be back:

*I think because all our rehearsing in the pandemic has been on Zoom, so we’ve been accustomed to only hearing our own voice and the voice of the musical director* (P3).

For most, however, the ability to attend events in-person was a positive change. Many participants expressed a feeling of elation and excitement related to their first live events after the easing of restrictions:

*Although it was very empty, I think that was the first show I went to with the theatres reopening. And that just felt like this is the start of something. And it was like, yeah, I’m taking my mom out and we are going somewhere. That affected me a bit more in a sort of feeling a bit weepy because you sort of think, this is what we used to take for granted* (P4).

Since the interviews took place after the 19th of July and covered the previous 3 months, the participants provided a rich account of their experiences during the transitional period when varying levels of restriction were still in place. Reported differences in experience of arts and cultural activity, when compared with pre-pandemic times, most often included shorter performances, limited facilities (bars and buffets were closed, so that participants could not stay for a drink afterward), needing to book a timed ticket (for art galleries), social distancing measures, mask-wearing requirements, and COVID-testing. These measures were mostly mentioned in a pragmatic way. Participants described them as a “needs must” and a minor factor in their overall experience. As the interviews progressed, however, it became clear that for some, their experience had been, in fact, negatively affected by these venue-related restrictions. The key factor that made a difference was the limited audience numbers which resulted in the event feeling less collective:

*I think the venues did a decent job. But it’s not*… *It’s not as exciting, as immediate as when you’ve got a lot more people there* (P1).

At the same time, some saw this as a positive aspect, creating the intimate atmosphere experienced at smaller venues with restricted audience numbers:

*The chairs were laid out, you know, in like, little groups, but there were more people there because it’s smaller, it was more of a cosier feeling anyway* (P1).

Some participants reported that the restrictions improved their experiences of in-person events in other ways. In particular, the requirement of a timed ticket in art galleries allowed for a more relaxing and less busy experience, avoiding queues and crowds:

*In some ways, it’s been nicer. Like the thing about booking to go to galleries and stuff, which you have to do now, is actually sometimes it works out better, because you know, there’s not going to be great big queues in front of something, or you’re not going to be waiting for ages. And so in some ways that’s nicer because you can plan things a little bit better* (P4).

Another positive aspect of the restrictions and, specifically, social distancing, was the enhanced quality of the activity because those engaged had to focus more and listen to each other. Here, one participant talks about attending a socially distanced choir rehearsal on a roof of a building:

*When we did the recordings on the rooftop, the quality of the singing, actually, okay, it was a smaller group, but the quality of the singing was better because people had to listen more to each other because they were distanced* (P6).

This participant also talks about a musical performance at the Liverpool Philharmonic and notes that the experience was more focused and allowed for fewer distractions:

*I also felt that in the Phil[harmonic], I know that it would have been difficult for the musicians initially, they have had to choose some of the repertoire, placed themselves in a different way than they were used to. But I wonder actually whether*… *I didn’t feel that the musical experience was lessened. In fact, I think it may well have been enhanced, because there’s a certain element where you have just got to really, really, really be so concentrated in the zone* (P6).

#### Managing change during the transitional period

The focus of this subtheme is the macro and external changes affecting audiences’ experiences, including alterations to restrictions, the fluctuating number of COVID cases, and the changing of the seasons. At the time of the interviews, many participants felt optimistic and hopeful about the future and were actively planning new events to attend in autumn and later in 2022:

*I couldn’t tell you exactly, but probably, even until the end of the year, I’ve got something like about twenty different events. More so music, and some theatre. Twenty in total. Because when I get going, you know, I could probably go to a gig every week. And 2022 is looking even more promising* (P1).

The overarching feeling expressed, however, was that of uncertainty. It is clear that participants continued to feel unsure about the pandemic and the possibility of lockdowns in the future:

*We can’t, I mean, I guess we can’t know, between now and September, in September, if the regulations alter again, everything’s uncertain* (P3).

Interestingly, some participants reported feeling more cautious at this point (in July 2021) than in the previous interviews in April 2021 (Chapple et al., under review):

*At the moment, definitely [*continue to be cautious*]. Much more so than I was a couple of months ago. A couple of months ago, yeah, I was going on the bus with a mask on, and I felt comfortable doing that. But I just don’t think there is a point at the moment until it’s clearer which way things are going* (P2).

It is also interesting to note how the notion of “normality” was discussed in the interviews. Some participants, for instance, mentioned being happy to “feel normal” again when attending in-person events:

*So yeah, it’s been nice sort of to start getting a bit closer to normal things in this sort of transition space* (P6).

Others, however, rejected the term “normal” altogether, commenting that it will probably not be possible to “go back to normal” for a long time now:

*I don’t think normal is the right term, actually, I don’t agree with people, saying we’ll go back to normal. It’s going to be, it’s just different. So hopefully, we can find joy in that different way of living* (P8).

#### Taking risks: Varying levels of safety

One of the most prominent subthemes that dominated many of the interviews was the risk-balancing inevitably associated with resuming in-person events. Risk assessment became an important part of returning to in-person events. At this time point (i.e., post 19 July), participants continued to talk about attending live indoor events as a risk:

*So, if you’re going to a big event, you think okay, well, essentially, I’ll take the risk, because this still feels like it’s a risk* (P5).

Other than the direct risk to their health and wellbeing, participants mentioned the risk of being absent from work and, as a result, struggling financially:

*Because there’s also work on top of that, it’s quite high pressured at the moment. And I don’t think it could withstand me being off for any period of time, from being what I would say, is being a little irresponsible really* (P8).

Another important factor considered by participants was how they would reach the venue. Many reported, in particular, that using public modes of travel was a stressful experience that they would prefer to avoid:

*However, more of an issue is getting there because normally I had to travel on public transport. And I don’t feel that comfortable being on public transport right now* (P2).

Taking into consideration all of the potential risks and concerns mentioned above, it is understandable that the return to in-person engagement was cautious and gradual. Some participants reported not feeling ready to return yet. Even those who had returned to in-person engagement, expressed wariness. One participant described a recent experience of going to the cinema and going for a coffee before and after the screening:

*And we met for a coffee in an outside cafe beforehand. And then we had coffee afterwards outside again. And I did notice that I enjoyed the second coffee much more than I enjoyed the first coffee. Because the first coffee I was*… *It was just a bit of kind of butterflies in my tummy. I suppose it was low-grade anxiety* (P5).

Looking forward, however, some participants felt optimistic and hopeful about returning to pre-pandemic levels of engagement, again describing the gradual transition to in-person events as a learning curve:

*But I do think that we’ll learn a bit more about how confident we’re feeling about going out and stuff. As the months go past, so maybe hopefully next year, I’ll start looking for things to go see again* (P5).

It is important to note that those who returned to events in-person before the 19th July with some restrictions in place, did feel safe at the events they attended:

*Of course, I felt quite comfortable there because it’s a big space. Because it was all distanced. [.] So there was plenty of room there* (P6).

The findings suggest that the patterns of engagement might change long-term and have implications for arts and culture organizations in the future. Specifically, many mentioned that, at least at this time point, some level of restrictions was preferable to no restrictions at all:

*Last Monday the restrictions were lifted. And funnily enough, although I really enjoyed being in the theatre, at the same time, I didn’t feel quite as safe. You know, because there wasn’t any space in between the seats* (P3).

*Actually, given that, obviously, things are still ongoing. And COVID cases are quite high. They actually made me feel more comfortable. So much so that I don’t know that I would do the same now, since restrictions have lifted* (P8).

Important measures that made the audience feel safer included social distancing, good air ventilation, and mandatory mask-wearing. Another factor was familiarity with the venue:

*And it felt safe, you know, I think because we used to go to Philharmonic fairly often, we might go six times in a season or something. So, we feel familiar with the surroundings* (P3).

*My brother went and I saw a picture of the auditorium and noticed that it was really spaced out. People had their masks on. The organisation as well was following correct procedures. And they took temperatures on the way in and things like that* (P7).

Many participants emphasized the importance of trusting the organization to ensure COVID-safety at the events they were attending:

*You go to organisations that you feel like you trust to follow guidelines to keep you safe* (P7).

Some also highlighted the importance of clear communication about the measures taken to instill a feeling of safety:

*I think the thing was that they had made very, very clear in the material around the concert, all the things that they were doing to keep people safe. And we knew what to expect, even though it felt sort of unusual compared to you know, what you might do normally, we were ready for it. And I think that was really helpful. So it didn’t feel like you were doing something strange in that space, it felt more like you were moving towards doing something closer to normal, because you’re able to be there* (P6).

Some suggested, looking ahead, that some measures taken by arts and cultural providers in response to COVID-19 might need to remain in place:

*Certain things are going to have to sort of change permanently. I imagine certain venues will need better ventilation or need better seating arrangements, and that kind of thing, really* (P8).

### Moving forward: Online and blended provision

#### Ongoing considerations around online inclusion

As discussed earlier, the return of the audience to in-person provision was gradual and cautious. It is evident in that case, that online or hybrid arts and cultural provision continued to be a vital option for many. At the same time, there were numerous concerns around the accessibility of, and some barriers to, online provision that continued to impact audiences’ experiences. In accordance with findings from previous waves of this study (Chapple et al., under review), participants preferred in-person provision to online if they had a choice. In particular, many noted that the social aspect of engaging in arts and culture was preferable and more enjoyable in person:

*I think when you’re face to face, you can tell when somebody is going to talk. And it just feels a lot more*… *Because we are used to it. So, it sounds a lot more comfortable being face to face and talking, sharing ideas. [*…*] Yes, it felt much nicer, much, much nicer than doing on Zoom* (P5).

Other practical barriers highlighted by participants included technical issues, cost, and difference in time zones. Similar to the interviews conducted with representatives from arts and cultural organizations within the LCR (Worsley et al., 2022), screen fatigue was among the factors cited as a barrier to online engagement:

*I’m online all day at work, all my meetings are online. Yeah, I’m by the tiny laptop screen all day, every day in the office. So yeah, that’s not necessarily something that I want to be doing more of outside of work* (P2).

In addition to these barriers to online engagement, some participants were keen to resume missed opportunities in-person as soon as it became possible. Summer season, better weather and longer days were decisive factors in favor of opting for in-person engagement:

*Now, at the moment, I just want to spend Summer doing things, going to things and trying things again* (P4).

Another common barrier to online engagement was the difficulty of connecting with others online. For instance, a participant talked about a recent experience of a Zoom workshop that was intended as a platform for different generations to come together. The participant, however, struggled because the younger participants refused to turn their cameras on:

*So, the intention was to bridge generations. And actually, it didn’t do that initially. Well, it didn’t do that, in some ways to me at all. Because I found it really hard to build a relationship with someone that I couldn’t see, and that never spoke* (P6).

The difficulty of connecting to others online was also mentioned by this theater practitioner who staged an online streamed theater production during lockdown:

*I’ve kind of almost distanced myself from that emotional experience. And it, it did feel a little bit like the whole event ended a little bit abruptly. There was no taking a bow or having a chat in the bar. It just felt a little bit of a let-down. And that was really hard* (P7).

The same participant, however, reported that having audience members respond by sending feedback (despite the time delay) made the experience more valuable. The participant added that, in a way, online feedback seemed more sincere than the immediate reactions they would usually get during a live performance:

*So, when I started getting messages to say the people have enjoyed it, and it has resonated with them, I found it quite emotional.*

<…>

*And then it felt a little bit more sincere. Because I think when you’re at a show, everyone says it is great, that’s great. But then to put that down in a message and explain why it felt like it had a bit of sincerity to it* (P7).

Finally, this subtheme also captures some new concerns around inclusion and exclusion, relating to accessibility for vulnerable people. When restrictions were lifted, online provision retained its importance, and vulnerable people continued to benefit from these hybrid opportunities.

Some participants also expressed concerns for vulnerable friends and family and awareness of putting them at risk when attending in-person events. An important aspect pointed out by participants was the responsibility associated with potentially putting others at risk when planning an event:

*Everything you do at the moment is about is what you’re doing worth the risk? And everything that somebody else suggests, it’s worth the risk, because somebody else’s suggested it and I want to be with them. But I am not quite ready myself to suggest that somebody else takes the risk with me if you see what I mean* (P6).

The importance of maintaining online and hybrid provision to ensure inclusivity, particularly in the colder months of the winter when the opportunity for outdoor events will be limited, was highlighted:

*And, you know, whatever happens in September, we’re ready to go inside again, in a bigger space, but it may well be that some of those people still feel vulnerable. So we might find ourselves continuing to do some alternatives on Zoom. I don’t know, we have to see how it goes. But what we don’t want to do is to exclude anybody, because they feel uncomfortable* (P6).

#### Navigating online content

This subtheme captures the changing experiences of arts and cultural engagement online and some practical issues with respect to online provision. One important aspect revealed in the interviews was the selective and more functional approach to hybrid provision among participants. In particular, they tended to use online resources to seek information about arts and cultural events. Specifically, participants preferred social media for these purposes, which might be an incentive for arts and cultural organizations to use their social media platforms more:

*Well, I did use some websites of institutions, some of them are good. But more and more I go to social media for information, so you know, on Facebook, you know, I’m more likely to see a link to whatever and then just go to websites, instead of going straight to the website and find where the thing is* (P1).

Online provision was regarded as preferable for professional development;

*Yes, sometimes I do actually [prefer online workshops to in-person], because it’s just a bit more convenient. And I think the only thing I want from these workshops is kind of information? It’s not like I’m going for the social experience or anything* (P7).

Whilst some arts and cultural activities were also regarded as more suited to online provision, others appeared more difficult to replicate online:

*So, the theatrical productions are good to watch online, but it can’t beat the experience of going to watch actors in the theatre. Cinema*… *I could take it or leave it. It’s easier watching it at home. Art, I would rather go to an art gallery to appreciate it, rather than looking at it online. So, I think online is good, in some ways. It’s filled a gap. But it’s only a small piece of it* (P1).

As these comments suggest, preference for online or in-person delivery varied by activity, and the selection of mode was dependent on individual preference. This participant, for example, argued that online provision might be less suitable for live music concerts:

*There is obviously a social aspect to going to a gig. So yeah, very different in that respect* (P2).

## Discussion

This study aimed to explore the changing experiences of in-person engagement in arts and culture after the lifting of the COVID-19 restrictions in July 2021 in the LCR. The findings suggest that the day the restrictions lifted was not a clear-cut return to “normality” for the participants. Instead, this change was the start of a new period of transition forging a “new normal” where the pandemic and pre-pandemic experiences were combined. Interestingly, the whole idea of “normality” has been transformed, and while some were eager to return back to “normal,” others rejected the idea altogether, arguing that the “norm” itself has changed during the pandemic. This, in turn, has opened up questions about the long-term effects of the pandemic on the arts and culture industry and the audience’s patterns of engagement in arts and culture in the months and possibly years to come.

The crucial role of arts and culture for the sense of self for the participants was highlighted. On resuming in-person engagement after a long break, participants noted that they were finally able to start rebuilding their sense of self previously negatively impacted by the pandemic. Our findings also demonstrate that arts and culture maintain their importance for personal development and growth. Engaging in arts and culture helped participants to cope with the stresses of this transitional period and such engagement became an important source of resilience. Attending arts and culture venues also helped the participants gradually rebuild the confidence to engage in other activities in person.

While the importance of social connectedness and a sense of belonging through arts and culture remains strong, several risk concerns surfaced in the interviews. Compared to our findings at waves (i) and (ii) (Chapple et al., under review), participants expressed increased anxieties, in the most recent period covered by the study, related to putting their friends and family at risk when attending in-person events.

The impact of the pandemic was also evident in the reframed cultural identity reported by some participants. The increased availability of online provision has in some ways blended the lines between local and global arts and culture. While participants reported a renewed appreciation and desire to support local arts and culture institutions, they were also exposed to a wider global variety of arts and culture, which broadened their horizons.

Undoubtedly, patterns of in-person engagement in arts and culture changed significantly during the pandemic. In line with recent research (e.g., The Audience Agency, 2021), our findings show that the return to in-person events proved to be slow and cautious. This transitional period was widely affected by risk-balancing directly linked to the safety measures taken by organizations. Some participants highlighted the importance of trusting the arts providers and being familiar with the measures taken in order to feel more confident when returning to in-person events.

While some reported that their experiences became more limited, many noted that the experience of arts and culture was actually enhanced by the COVID-19 restrictions, making it easier to focus on the art and easier to avoid distractions. Social distancing and timed entries also positively affected the cultural experience for some.

The findings suggest that online provision remains vital for many, ensuring wider inclusivity, particularly for vulnerable audiences. Since many still viewed attending in-person events as a risk, participants appreciated the option of alternative provision as the pandemic continued to unfold. At the same time, it is important to acknowledge the potential barriers to online inclusion and the possibility of a growing digital divide. Some participants mentioned the cost and accessibility of online provision coupled with screen fatigue as barriers to online engagement in arts and culture. Online provision, these findings suggest, may also struggle to replicate the feeling of connectedness experienced during in-person live events.

For arts organizations, moving forward, it will be important to gather the audience’s feedback as the patterns of engagement in arts and culture continue to change as a result of the pandemic, and to consider the channels of communication used to connect to potential target audiences. For example, attention to the clarity of communication on safety measures, setting out clear rules and ensuring that audiences are familiar with them, will mitigate some of the anxieties that audiences feel when they balance risks in relation to returning to in-person events. On the other hand, while audiences are keen to support local arts and culture, a renewed interest in traveling for arts might open up new opportunities to reach wider groups of beneficiaries.

Finally, it is crucial to note that, despite some limitations, digital provision addressed some of the audience’s risk and safety concerns in this transitional period and beyond. Based on the beneficiaries’ responses, online experience could be enhanced by further prioritizing and encouraging social interaction, putting additional effort into facilitating discussions and encouraging input from all participants.

There are several limitations to this study that should be considered. First, this study includes a relatively small sample of participants and the time period it covers does not go beyond August 2021. In the future, it would be important to look at the changes in subsequent months in order to evaluate any long-standing effects of the pandemic on the audience’s experience. Second, this study is geographically limited to the LCR. It would be useful to explore patterns of engagement in different regions of England and beyond in order to get a sense of arts and cultural engagement at national level. Third, it might be useful to look specifically at the experiences of vulnerable people in the LCR. Finally, it would be useful to dedicate future research to identifying in more detail the preferences toward online and offline provision for specific types of arts and culture events.

To conclude, this study has highlighted the benefits of engaging in arts and culture during the COVID-19 pandemic. As the findings suggest, the role of arts and culture remains crucial in supporting mental health and wellbeing in the LCR. The findings support previous research showing that engagement in arts and culture during challenging and transitional times can help reduce stress and increase the ability to cope with traumatic experiences. Importantly, the study shows that engagement in arts and culture was beneficial not only during full lockdown (Chapple et al., under review) but also during the more recent transitional period of return to in-person engagement. This might suggest that engagement in arts and culture will continue to be crucial in the immediate future and beyond, possibly becoming one of the vital tools of processing the collective trauma of the COVID-19 pandemic.

## Data availability statement

The raw data supporting the conclusions of this article will be made available by the authors, without undue reservation.

## Ethics statement

The studies involving human participants were reviewed and approved by University of Liverpool Research Ethics Committee (reference: 7994). The patients/participants provided their written informed consent to participate in this study.

## Author contributions

JB and EB conceived the study. AA collected the qualitative data, analyzed the qualitative data to finalize the framework for wave 3, in consultation with the research team, and wrote the first draft of the manuscript. AA and MC coded a subset of transcripts. MW and MC analyzed wave 1 and 2 data and created a working thematic framework that informed the wave 3 framework. JB, EB, JW, and MW read, commented on, and revised the manuscript providing important intellectual input. All authors have read and approved the final version.

## Footnotes

1 Participant.
