# Supplementary material for: Back to live: Returning to in-person engagement with arts and culture in the Liverpool City Region
Source: Front Psychol. 2022 Oct 26;13:1011766. doi: 10.3389/fpsyg.2022.1011766 (PMC9643399; doi:10.3389/fpsyg.2022.1011766)
Supplement: Supplementary file 1 [file Data_Sheet_1.DOCX]

Supplementary Material

# (Qualitative) Interview Schedule

1. **Welcome and Introduction (5 mins approx.)**
2. **Arts and Culture engagement (10-15 mins approx.) Thinking about the last two months.**
3. How did the COVID-19 lockdown period have an impact upon your current engagement with arts and cultural activity in the Liverpool City Region?
4. Which kind of arts and cultural activity in the Liverpool City Region did you engage in?
5. What motivated you to participate in arts and cultural activity in the Liverpool City Region?
6. Which arts and cultural activities did you most enjoy and why?
7. Which arts and cultural activities did you most miss and why?
8. How did you get access to arts and cultural activity in the Liverpool City Region during the last two months?
9. Did you find some activities easier/more difficult to access than others? If so, which ones?
10. **Changing experiences (5-10 mins approx.) Thinking about the most recent full lifting of restrictions (July 2021).**
11. How has your experience of arts and cultural activity in the Liverpool City Regions changed since the full lifting of restrictions? (How far have you resumed former activities? Or continued with those taken up during lockdown?)
12. What are your plans and thoughts on arts and cultural activity in the future?
13. Is there anything else you would like to say?
14. **Thank you and debrief (5 mins approx.)**
